# A Remarkably High-Speed Solution-Phase Combinatorial Synthesis of 2-Substituted-Amino-4-Aryl Thiazoles in Polar Solvents in the Absence of a Catalyst under Ambient Conditions and Study of Their Antimicrobial Activities

**DOI:** 10.5402/2011/434613

**Published:** 2011-04-11

**Authors:** Satish N. Dighe, Pratip K. Chaskar, Kishor S. Jain, Manisha S. Phoujdar, Kumar V. Srinivasan

**Affiliations:** Department of Pharmaceutical Chemistry, Sinhgad College of Pharmacy, Vadgaon (Bk), Pune, Maharashtra 411041, India

## Abstract

Remarkably high-speed synthesis of 2-substituted amino-4-aryl thiazoles in polar solvents with a minimum threshold polarity index of 4.8 was found to proceed to completion in just 30–40 sec. affording excellent yields of thiazoles under ambient temperature conditions without the use of any additional catalyst. The purification-free procedure afforded libraries based around a known pharmacophore, namely, substituted arylthiazoles and generated samples of high purity. In terms of combinatorial synthesis in a single solution phase, our protocol is significantly better than those hitherto reported and is amenable for HTS. The *in vitro* biological tests of some thiazoles showed good activity towards gram-positive bacteria, gram-negative bacteria and fungi comparable with the standard drugs, nitrofurantoin and griseofulvin, for their antibacterial and antifungal activities, respectively.

## 1. Introduction

Thiazole and its derivatives are very useful compounds in various fields of chemistry including medicine and agriculture. For example, the thiazolium ring present in vitamin B1 serves as an electron sink, and its coenzyme form is important for the decarboxylation of *α*-keto acids [1]. This heterocyclic system has found broad applications in drug development for the treatment of inflammation [2], hypertension [3], bacterial [4], and HIV infections [5]. Aminothiazoles are known to be ligands of estrogen receptors [6] as well as a novel class of adenosine receptor antagonists [7]. Other analogues are used as fungicides, inhibiting *in vivo* growth of Xanthomonas, as an ingredient of herbicides or as schistosomicidal and anthelmintic drugs [8]. 

In view of the importance of 2-aminothiazole and its derivatives, several methods have been reported in the literature. Hantzsch reaction of *α*-halocarbonyl compounds with thioureas or thioamides provides a useful method for the synthesis of thiazoles [9]. Solid-supported syntheses have been used to generate small organic libraries [10] and solution-phase preparation of combinatorial libraries has been reported in DMF [11]. Recently, many improved methods have been reported for the synthesis of thiazoles using catalysts such as ammonium molybdophosphate (AMP) in methanol [12], *β*-cyclodextrin in water [13], Iodine [14] and by the use of microwave in ethanol [15]. However, in spite of their potential utility, many of these reported methods suffer from some drawbacks such as harsh reaction conditions, unsatisfactorily yields, cumbersome product isolation procedures and the use of expensive catalysts. These processes also generate waste-containing solvent and catalysts, which have to be recovered, treated and disposed off. The development of efficient and environmentally friendly chemical processes for the preparation of biologically active molecules constitutes a major challenge for chemists in organic synthesis. In this context, in recent times, room-temperature ionic liquids (RTILs), especially those based on the 1,3-dialkylimidazolium salts, have shown great promise as an attractive alternative to conventional solvents (e.g., VOCs). They possess the unique advantages of high thermal stability, negligible vapour pressure, immiscibility with a number of organic solvents and recyclability [16–19]. In many cases, the products are weakly soluble in the organic phase so that the products can be easily separated by simple extraction. Very recently Dighe et al. have investigated the synergy of the combined use of ionic liquid and DMSO in the proportion 0.1 : 1 to synthesize a variety of esters in remarkably short reaction times from acyl or alky halides by their reaction with sodium carboxylates in the above mixed solvent medium in the absence of any added catalyst under ambient conditions. In individual solvents, there was no reaction of phenacyl bromide with sodium benzoate under ambient conditions in DMSO whereas that in the IL took 16 h for completion under similar conditions [20]. Using the above solvent conditions, a remarkably convenient and rapid synthesis of tetrazoles has been achieved recently by the same authors [21].

 We extended the investigation of this solvent system towards the synthesis of 2-aminothiazoles by the reaction of phenacyl bromides **1** with thiourea **2** at room temperature which afforded the corresponding 2-substitutedamino-4-aryl thiazoles derivative **3** in excellent isolated yields in remarkably short reaction times within 30–40 sec. Recently, Potewar et al. have reported the synthesis of these 2-aminothiazoles by using ionic liquid which was completed within just 15 minutes [22]. Potewar et al. [23] have also reported the synthesis of these thiazoles in water and the reaction was completed within 90 minutes. It is pertinent to add here, in water, phenacyl bromide was found to be insoluble and thiourea is soluble so that a slurry phase reaction phase resulted in increased time. In alcohol, however, both phenacyl bromide and thiourea are soluble so that the single-phase reaction required very less time. All these procedures make use of environmentally benign processes.

## 2. Results and Discussion

**Scheme 1:**
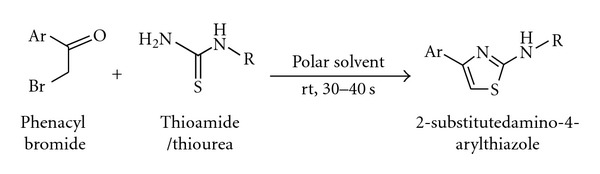


The reaction of phenacyl bromides **1** and thioamide or thiourea **2 **at ambient temperature in the presence of DMSO: IL medium (1 : 0.1) afforded the corresponding 2-amino-4-arylthiazole derivatives **3** in excellent isolated yields within 30–40 seconds (Scheme 1).

In order to highlight the role of DMSO in the scheme, various other dipolar aprotic solvents were also screened along with the IL in the proportion of 1 : 0.1 for the model reaction of phenacyl bromide with thiourea. The results are recorded in Table 1. 

It was found that under similar conditions, all the dipolar aprotic solvents afford the products in the same yields and reaction times as was found for the DMSO: IL system. It was then surmised whether IL is required at all in the reaction with the dipolar aprotic solvents individually contributing to the minimum polarity index of the media responsible for inducing the reaction. To our surprise, all dipolar aprotic solvents individually also gave excellent yields in just 30–40 sec. The performances of the solvents are recorded in Table 2. 

Herein, an assumption was made that a solvent with a minimum threshold polarity index [24] should be able to induce the reaction. In order to test this assumption, the reaction of phenacyl bromide with thiourea was performed in both dipolar aprotic solvents and protic solvents such as methanol, ethanol and *n*-butanol with varying polarities in addition to the nearly nonpolar CCl_4_. Results are recorded in Table 2. Extremely rapid reactions in 30–60 sec were observed in the solvents in the polarity index range of 4.1–7.2 whereas reactions in *n*-butanol and dichloromethane with polarity index in the range of 3-4 could be completed in reaction time of 1.5–4 min. No reaction was observed in nonpolar CCl_4_ even after 30 min. 

**Figure 1:**
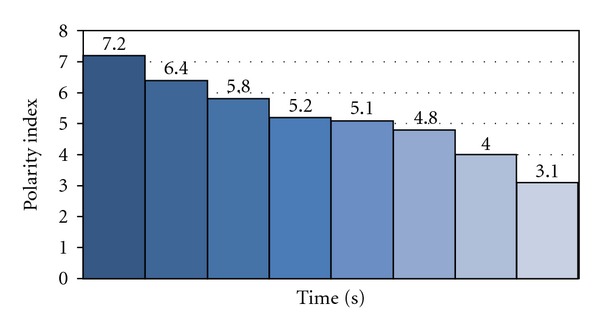
Time for completion of the reaction versus polar index.

Thus, after having examined the scope of this method in various solvents, since DMSO afforded the best results, all further reactions were carried out in DMSO as solvent. To begin with on a smaller scale (50 mg scale), we had taken out sample from the reaction mixture at the end of 30, 40, 50, 60, 90, 120, 150, 180, 210, 240 sec using a stopwatch in the initial phase and quenched the reaction in ice to confirm at which time reaction was completed. It was concluded that the reactions were completed in majority of the cases in just 30–40 sec. Further for consistency in results the reactions were scaled up to 100 mg and then to 1.0 g scale which has been reported. The results obtained were plotted, that is, time for completion of the reaction 30–180 sec versus polarity indices 3.1–7.2 for the same model reaction as shown in Figure 1. The time for completion of the reaction more or less decreases proportionately with decreasing polarity indices.

Several phenacyl bromides consisting of both electron withdrawing and electron-donating groups reacted smoothly with thiourea or thioamide in DMSO as solvent to give 2-substitutedamino-4-aryl thiazoles in 91–96% yields within 30–40 sec under ambient reaction conditions. The results are summarized in Table 3. It can be seen that the process tolerates both electron-donating and electron withdrawing substituents in phenacyl bromides. In all cases, except for the reactions of phenacyl bromides with an electron-donating methyl group, the reactions proceed very efficiently at ambient reaction temperature to afford the corresponding thiazoles within 30–40 sec in excellent isolated yields. For phenacyl bromides with an electron-donating group such as methyl, the reactions took 180 sec instead of 30–40 sec affording the same excellent yields. In combinatorial synthesis of 2-aminothiazole in the solution-phase synthesis in a recent report, the authors have reported the time for reaction to be 5h at 70°C in anhydrous DMF as solvent [25]. The solution-phase synthesis libraries that can be proposed by our protocol is much improved with significantly smaller reaction periods (30–180 sec) and important for HTS due to the remarkably rapid reaction periods (30 sec at 30°C as compared to 5 h at 70°C in the previous report). Among these 25 compounds, compound nos. **3a**, **3b**, **3e**, **3f**, **3h**, **3i**, **3o**, **3t**, **3u **having reported mp in literature which are nearly similar to our synthesized compounds. 

The above protocol was extended to synthesis of fanetizole **3d** an anti-inflammatory agent reported to have reached phase III clinical trial for the treatment of rheumatoid arthritis [26, 27]. The procedure designed herein can be so readily be extended towards the preparation of Fanetizole, for instance, using ethanol as solvent with the necessary polarity index to evolve an environmentally friendly process that serves to illustrate the potential of this methodology for manufacturing processes and to generate libraries which contain biologically active molecules. Moreover, the process described here requires little specialized knowledge whereas various reported solid-supported methodologies requires the practicing medicinal chemist to have knowledge of specialized techniques. In all the cases, the reaction was quenched by drowning into ice-water at the end of 30–40 sec and 180 sec, respectively. The time monitoring was done by a stopwatch.

### 2.1. Antimicrobial Activity

All the compounds were tested *in vitro* for their antibacterial and antifungal activity. The microorganisms used in this study were gram-positive bacteria, gram-negative bacteria and Fungi. These compounds were compared with standard drugs, Nitrofurantoin and Griseofulvin for their antibacterial activity and antifungal activities, respectively. Among all tested compounds, **3e, 3k, 3m, 3q, 3s** showed good antifungal activity and antibacterial activity against both gram positive and gram negative bacterial species. Therefore MIC's of these compounds were only evaluated and found comparable with those of the standard antifungal and antibacterial drugs (Tables 4 and 5).

## 3. Experimental Section

### 3.1. Microbiological Analysis

Micro-organisms used in this study were as follows: gram-negative bacteria *Acinetobacer lowfii*,* Pseudomonas aurogenosa, *gram-positive bacteria* Bacillus subtilis, Staphylacoccus aureus *and Fungi *Fusarium oxysporum, Aspergillus parasiticus, Aspergillus fumigates, Alternaria solani*. 

Antimicrobial activity was examined by the disc diffusion method under standard conditions using Mueller-Hinton II agar medium (Becton Dickinson) for bacteria and Potato-dextrose agar for fungi (according to CLSI guidelines) [28, 29]. Sterile filter paper discs (5 mm diameter, Whatman no. 3 chromatography paper) were dripped with compound solutions (DMSO) to load 500 mg of a given compound per disc. Dry discs were placed on the surface of appropriate agar medium. The results for bacteria (diameter of the growth inhibition zone) were read after 18 h of incubation at 36°C and for fungi were read after 30 h of incubation at 36°C. Compounds which showed activity in disc diffusion tests were examined by the agar dilution method to determine their MIC's minimal inhibitory concentration (CLSI) [30, 31]. The MIC's of the compounds were studied by disc diffusion method making serial dilution of the compounds from the range 2–2048 *μ*g/ml. The final inoculums of all studied bacterial organisms were 10^4^ CFU ml^−1^(colony forming units per ml). For fungal cultures, optical densities of spores in 0.2% tween 80 solutions were adjusted to 50 at 540 nm using colorimeter.

### 3.2. General Procedure

In a typical reaction, a mixture of phenacyl bromide (1 gm, 3.6 mmol), thiourea (0.29 gm, 3.6 mmol) in 10 ml of DMSO was stirred at room temperature until completion of the reaction. The time of the reaction was monitored by a stopwatch. The progress of the reaction was monitored by thin-layer chromatography. On completion of the reaction, the reaction mixture was drowned into crushed ice. The precipitated product was filtered and dried. The product was pure enough (single spot on TLC) for all practical purposes. However, for characterization purposes, it was further purified by column chromatography. The yield of the dried product was found to be 0.96 g (96%).

### 3.3. Combinatorial Synthesis

In the parallel synthesizer wherein 10 samples can be stirred simultaneously, the products (**3a**–**3y**) were simultaneously synthesized starting from respective raw materials consisting of substituted phenacyl bromides and thioureas/thioamides taking care to quench the reaction by addition of ice-water after 30–40 sec and 180 sec, respectively. 

Characterization of representative compounds is given as below.

#### 3.3.1. 4-Phenylthiazol-2-Amine (3a)

IR (KBr) 3433, 3019, 1602, 1531, 1519, 1482, 757 cm^−1^; ^1^ *H* NMR (200 MHz, CDCl_3_): *δ* 5.87 (bs, 2*H*. N*H *_2_), 6.48 (s, 1*H*, thiazole *H*), 7.02–7.20 (m, 3*H*, Ar*H*), 7.54–7.59 (m, 2*H*, Ar*H*); Anal. Calcd for C_9_H_8_N_2_S: C, 61.34; H, 4.58; N, 15.90%. Found: C, 61.43; H, 4.46; N, 15.81%.

#### 3.3.2. N_4_-Diphenylthiazole-2-Amine (3b)

IR (KBr) 3404, 3019, 1601, 1599, 1541, 1498, 1311, 758 cm^−1^; ^1^ *H* NMR (200 MHz, CDCl_3_): *δ* 6.83 (s, 1*H*, thiazole *H*), 7.02–7.11 (m. 1*H*, Ar*H*), 7.30–7.44 (m, 7*H*, Ar*H*), 7.49 (bs, 1*H*, N*H*), 7.83–7.87 (m, 2*H*, Ar*H*); Anal. Calcd for C_15_H_12_N_2_S: C, 71.40; H, 4.79; N, 11.10%. Found: C, 71.54; H, 4.8; N, 11.22%.

#### 3.3.3. N-Phenethyl-4-Phenylthiazol-2-Amine (3d)

IR (KBr) 3196, 3016, 2975, 1602, 1584, 1552, 1495, 1463, 1335, 754 cm^−1^; ^1^ *H* NMR (200 MHz, CDCl_3_): *δ* 2.94–3.01 (t, 2*H*, CH_2_), 3.52–3.62 (q, 2*H*, N–CH_2_), 5.24 (brs,1*H*, N*H*), 6.70 (s, 1*H*, thiazole *H*), 7.24–7.37 (m, 8*H*, Ar*H*), 7.76–7.80 (m, 2*H*, Ar*H*); Anal. Calcd for C_17_H_16_N_2_S: C, 72.82; H, 5.75; N, 9.99%. Found: C, 72.68; H, 5.81; N, 10.04%.

#### 3.3.4. N_4_-Bis(4-Chlorophenyl)-N-(Chlorophenyl)Thiazol-2-Amine (3k)

IR (KBr) 3335, 2923, 1588, 1520, 1495, 1482, 1399, 719 cm^−1^; ^1^ *H* NMR (200 MHz, CDCl_3_): *δ* 7.68 (bs, 1*H*, N*H*), 6.92 (s, 1*H*, thiazole *H*), 7.24–7.66 (m, 2*H*, Ar*H* of N*H*Ar-Cl), 7.55–7.98 (m, 2*H*, Ar*H* of Ar-Cl); Anal. Calcd for C_17_H_16_N_2_S: C, 56.09; H, 3.14; N, 8.72%. Found: C, 56.21; H, 5.3.05; N, 8.83%.

#### 3.3.5. 4-(4-Bromophenyl)-N-(4-Chlorophenyl)Thiazol-2-Amine (3q)

IR (KBr) 3383, 2934, 1597, 1524, 1552, 1492, 1398, 1338, 747 cm^−1^; ^1^ *H* NMR (200 MHz, CDCl_3_): *δ* 2.34 (s, 3*H*, Ar-CH_3_), 7.32 (bs, 1*H*, N*H*), 6.77 (s, 1*H*, thiazole *H*), 7.15–7.42 (m, 4*H*, Ar-*H* of Ar-CH_3_); 7.53–7.77 (m, 5*H*, Ar-*H* of Ar- Br); Anal. Calcd for C_17_H_16_N_2_S: C, 55.66; H, 3.80; N, 8.11%. Found: C, 55.78; H, 3.79; N, 8.14%.

#### 3.3.6. N_4_-di-p-Tolylthiazol-2-Amine (3w)

IR (KBr) 3437, 2923, 1614, 1570, 1534, 1501, 1381, 1333, 758 cm^−1^; ^1^ *H* NMR (200 MHz, CDCl_3_): *δ* 2.38 (s, 6*H*, Ar-CH_3_), 7.39 (bs, 1*H*, N*H*), 6.57 (s, 1*H*, thiazole *H*), 7.21–7.51 (m, 8*H*, Ar-*H* of N*H*-Ar-CH_3 _& Ar-CH_3_); Anal. Calcd for C_17_H_16_N_2_S: C, 77.82; H, 5.75; N, 9.99%. Found: C, 77.92; H, 5.75; N, 9.96%.

#### 3.3.7. 4-p-Tolyl-N-(4-Flurophenyl)Thiazol-2-Amine (3y)

IR (KBr) 3244, 2923, 1604, 1519, 1504, 1458, 1437, 1317, 731 cm^−1^; ^1^ *H* NMR (200 MHz, CDCl_3_): *δ* 2.40 (d, 3*H*, Ar- CH_3_), 7.79 (bs, 1*H*, N*H*), 6.69 (s, 1*H*, thiazole *H*), 7.16–7.48 (m, 8*H*, Ar-*H* of Ar-CH_3_), 7.74–8.08; Anal. Calcd for C_17_H_16_N_2_S: C, 71.40; H, 4.79; N, 11.10%. Found: C, 71.56; H, 4.70; N, 11.18%.

## Figures and Tables

**Table 1:** Screening of IL in combination with various dipolar aprotic solvents.

Sr. no.	IL+ dipolar aprotic solvents	Reaction time (sec)
1	(bbim)^+^Br + DMSO	30–40
2	(bbim)^+^Br + DMF	30–40
3	(bbim)^+^Br + acetonitrile	30–40
4	(bbim)^+^Br + Dioxane	60

**Table 2:** Screening of solvents for reaction.

Sr. no.	Solvents	Polarity index	Reaction time
1.	DMSO	7.2	25–30 sec
2.	DMF	6.4	30–40 sec
3.	Acetonitrile	5.8	30–40 sec
4.	Ethanol	5.2	60 sec
5.	Methanol	5.1	60 sec
6.	Dioxane	4.8	60 sec
7.	n-butanol	4.0	90 sec
8.	Dichloromethane	3.1	4 min
9.	Carbon tetrachloride	1.6	Not going even after 30 min

**Table 3:** Synthesis of 2-substitued-amino-4-aryl thiazoles.

Ar	R	Thiazoles	Time (Sec.)	Yield^a^ (%)
C_6_H_5_	H	**3a**	30–40	96
C_6_H_5_	C_6_H_5_	**3b**	30–40	93
C_6_H_5_	CH_3_	**3c**	30–40	92
C_6_H_5_	CH_2_CH_2_C_6_H_4_	**3d (Fanetizole)**	30–40	91
C_6_H_5_	4-Cl C_6_H_4_	**3e**	30–40	92
C_6_H_5_	4-NO_2_ C_6_H_4_	**3f**	30–40	92
C_6_H_5_	4-F C_6_H_4_	**3g**	30–40	93
4-Cl C_6_H_4_	H	**3h**	30–40	94
4-Cl C_6_H_4_	C_6_H_5_	**3i**	30–40	93
4-Cl C_6_H_4_	CH_3_	**3j**	30–40	91
4-Cl C_6_H_4_	4-Cl C_6_H_4_	**3k**	30–40	92
4-Cl C_6_H_4_	4-NO_2_ C_6_H_4_	**3l**	30–40	92
4-Cl C_6_H_4_	4-F C_6_H_4_	**3m**	30–40	94
4-Br C_6_H_4_	H	**3n**	30–40	95
4-Br C_6_H_4_	C_6_H_5_	**3o**	30–40	92
4-Br C_6_H_4_	CH_3_	**3p**	30–40	91
4-Br C_6_H_4_	4-Cl C_6_H_4_	**3q**	30–40	94
4-Br C_6_H_4_	4- CH_3_C_6_H_4_	**3r**	30–40	92
4-Br C_6_H_4_	4-F C_6_H_4_	**3s**	30–40	93
4-CH_3_C_6_H_4_	H	**3t**	180	93
4-CH_3_C_6_H_4_	C_6_H_5_	**3u**	180	94
4-CH_3_C_6_H_4_	CH_3_	**3v**	180	94
4-CH_3_C_6_H_4_	4-CH_3_ C_6_H_4_	**3w**	180	91
4-CH_3_C_6_H_4_	4-Cl C_6_H_4_	**3x**	180	93
4-CH_3_C_6_H_4_	4-F C_6_H_4_	**3y**	180	92

**Table 4:** Antibacterial activity of compounds with standard (diameter of the growth inhibition zone (Giz, mm) and minimal inhibitory concentration (MIC, mg/ml).

Strain	Compounds											
	3e		3k		3m		3q		3s		Nitrofurantoin	
	Giz	MIC's	Giz	MIC's	Giz	MIC's	Giz	MIC's	Giz	MIC's	Giz	MIC's
	(mm)	(*μ*g/ml)	(mm)	(*μ*g/ml)	(mm)	(*μ*g/ml)	(mm)	(*μ*g/ml)	(mm)	(*μ*g/ml)	(mm)	(*μ*g/ml)
*A. lowfii*	10	2	10	32	12	64	13	64	15	32	10	32
*P. aurogenosa*	10	4	13	2	10	32	10	64	12	32	11	32
*B. subtilis*	14	4	12	2	13	32	12	32	10	64	11	64
*S. aureus*	13	4	12	2	14	16	14	64	11	64	13	64

**Table 5:** Antifungal activity of compounds with standard (diameter of the growth inhibition zone (Giz, mm) and minimal inhibitory concentration (MIC, mg/ml)).

Strain	Compounds											
	3e		3k		3m		3q		3s		Griseofulvin	
	Giz	MIC's	Giz	MIC's	Giz	MIC's	Giz	MIC's	Giz	MIC's	Giz	MIC's
	(mm)	(*μ*g/ml)	(mm)	(*μ*g/ml)	(mm)	(*μ*g/ml)	(mm)	(*μ*g/ml)	(mm)	(*μ*g/ml)	(mm)	(*μ*g/ml)
*F. oxysporam*	12	16	7	8	13	32	10	256	11	16	9	16
*A. parasiticus*	11	64	8	64	11	64	10	128	12	256	8	32
*A. fumigates*	13	128	7	128	12	8	8	128	13	256	9	32
*A. solani*	10	8	6	32	11	128	7	32	13	8	9	64
